# The prevalence of frailty and its effect on the outcome in cardiac resynchronization therapy patients

**DOI:** 10.1007/s11357-023-01023-w

**Published:** 2023-12-21

**Authors:** Luca Katalin Kuthi, Walter Richard Schwertner, Boglárka Veres, Eperke Dóra Merkel, Richard Masszi, Anett Behon, Attila Kovács, István Osztheimer, Endre Zima, Levente Molnár, László Gellér, Annamária Kosztin, Béla Merkely

**Affiliations:** Heart and Vascular Center, Semmelweis University, 68 Városmajor Street, Budapest, 1122 Hungary

**Keywords:** Frailty, Frailty score, Frailty index, Cardiac resynchronization therapy, Heart failure

## Abstract

Frailty is a complex clinical syndrome associated with aging and comorbidities, which correlates with unfavorable outcomes. However, in heart failure patients, frailty is very common, data is scarce about those, who are eligible for Cardiac Resynchronization Therapy (CRT) implantation. We investigated the incidence of frailty and the association of Frailty Index (FI) with the outcome. Thirty baseline clinical parameters were used by the Rockwood cumulative deficit method to determine patients' FI in our single-center cohort. Based on previous studies, patients with FI ≤ 0.210 were considered as non-frail, those with FI 0.10–0.210 were classified in Frail-1, with FI > 0.10 in Frail-2 groups, respectively. Echocardiographic response after 12 months and all-cause mortality were investigated by frailty groups. Among 1004 included patients, 75 (7%) were considered Non-frail, 271 (27%) grouped in Frail-1, and 658 (66%) in Frail-2 with a median FI of 0.36 (0.28–0.43). Patients in Frail-2 group were older, with more comorbidities compared with non-frail patients or those in Group Frail-1. During the median follow-up time of 4.8 years, 29 (39%) patients died in the Non-frail, 140 (52%) in Frail-1, and 471 (72%) in the Frail-2 groups (log-rank *p* < 0.001). Group Frail-2 showed an unfavorable outcome compared to the non-frail (HR 2.49, 95%CI 1.92–3.22; *p* < 0.001) and the Frail-1 group (1.83, 95%CI 1.55–2.16; *p* < 0.001). In our HFrEF patients eligible for CRT implantation, patients were exceedingly vulnerable with a high prevalence of frailty. The calculated frailty index was associated with outcome and proved to be prevalent in individual risk stratification.

## Introduction

Frailty is a complex clinical condition that results from an aggregation of insults across multiple organ systems [1]. Frailty can be quantified by counting the number of ‘health deficits’ across a range of domains. Frailty is related to, but distinct from, both aging and comorbidities [2, 3]. The relationship between frailty and heart failure (HF) is of particular interest because these conditions often coexist, and each increases the likelihood of the other. Thus, patients with HF are up to six times more likely to be frail than the general population, and, due to shared pathophysiological mechanisms, HF may accelerate the development of frailty, and frail persons may be at higher risk for developing HF [4–7]. Frail patients with HF also have a substantially higher risk for death, hospitalizations, and functional decline than non-frail patients with HF. Reducing the risk for developing frailty, slowing its progression, and even reversing frailty are now recognized goals in the management of HF [8–14].

The subgroup of heart failure patients with reduced ejection fraction (HFrEF) and wide QRS is a selected patient population, that can show mortality and morbidity benefit after cardiac resynchronization therapy (CRT) implantation [15]. For those patients, who are proved to be responders, frailty can be modified and patients can be shifted to a better frailty class with an improved clinical state and organ functions [16]. However, the assessment and risk stratification by the patients’ clinical condition would be essential before CRT implantation, data about the long-term outcome by frailty is scarce in this cohort.

The aim of this study was to assess the prevalence of frailty in HFrEF patients eligible for CRT implantation from a real-world cohort, to classify them by severity using the Rockwood frailty method and to investigate its correlation with their long-term outcome. Additionally, to identify the most vulnerable patients whose management should be optimized.

## Methods

### Patient population

We retrospectively collected 2923 patients, who underwent successful CRT implantation at the Heart and Vascular Center of Semmelweis University between June 2000 and December 2020. As per the current guidelines those HFrEF patients with wide QRS (> 130 ms), symptoms (NYHA II-IVa), and lower left ventricular ejection fraction (LVEF) < 35% were implanted.

### Frailty index

By using the original Rockwood method [17] we constructed a 30-item frailty index to identify frail patients. The 30 items were derived from medical history, other patient characteristics, and laboratory results (Table 1). Binary variables were scored 0/1 (absent/present); ordinal variables were scored from 0–1, 1 indicating the greatest severity. Continuous variables were dichotomized and scored as 0/1 (normal/non-normal). Patients with ≥ 20% missing variables were excluded from the analysis. FI score was calculated using the recommended approach, i.e., the sum of the deficits divided by the total number of non-missing deficits assessed. In keeping with previous studies, patients with FI ≤ 0.210 were classified as non-frail based on conventional cutoffs used by most authors; those with higher scores were further divided into two categories using increments in a score of 0.1.

**Table 1:** Parameters for frailty score

Vital status
Diastolic Blood Pressure
Systolic Blood Pressure
Pulse pressure
BMI
NYHA
Laboratory parameters
BUN
Creatinine
Sodium
Total Cholesterol
Uric acid
Haemoglobin
Ly (%)
Potassium
Co-morbidities
AF
HT
MI
PCI/CABG
DM
COPD
Anaemia
CKD
Ventricular arrythmia
CIED
Cancer
Stroke
PAD
Echocardiographic parameters
LVEF
LVEDD
LVESD
TAPSE

### Optimal medical and device therapy and response to CRT

We assessed the proportion of nonresponders and patients on optimal medical therapy in each frailty group. We also evaluated the ratio of CRT-pacemaker (CRT-P) or CRT with a defibrillator (CRT-D) by frailty groups. We defined responders when a relative 15% improvement in left ventricular ejection fraction (LVEF) could be observed in 12 months after CRT implantation assessed by echocardiography, which was performed by an experienced certified echocardiography specialist. Optimal medical therapy was considered complete if the patient was on a beta-blocker, ACE-inhibitor, Angiotensin II receptor blocker or ARNI, and mineralocorticoid receptor antagonist at the same time.

The type of the CRT devices (CRT-P vs. CRT-D) was assessed individually by the implanting physician as per the current guidelines, preferably using CRT-D for ischemic patients, males, younger patients or those who were expected to be non-responders.

### Outcome data

The primary endpoint of the study was all-cause mortality. Patients’ status (dead or alive), and date of death were obtained for all patients by querying the National Health Insurance Database of Hungary in April 2022. The time to death was measured from the date of the CRT implantation.

Response to CRT was assessed 12 months after the implantation by echocardiography. Those more than 15% reative LVEF increase were considered as responders.

### Statistical analysis

Continuous variables are expressed as mean ± standard deviation (SD) or median and interquartile range (25^th^-75^th^). Statistical tests employed were unpaired Student’s t-test or Mann–Whitney U test or Spearman correlation (for continuous variables) and Chi-squared or Fisher’s exact test (for categorical variables), respectively. The survival of subgroups was visualized using Kaplan–Meier curves, and Log-rank tests were performed for comparison. Univariable and multivariable Cox proportional hazards models were used to compute hazard ratios (HR) with 95% confidence intervals (95% CI). A two-sided p-value of < 0.05 was considered statistically significant. All statistical analysis was performed in SPSS (version 25.0, IBM, Armonk, NY, USA), GraphPad Prism (version 8, Inc., GraphPad Software, SanDiego, CA, USA), and RStudio (version 1.8, RStudio PBC, Boston, MA, USA).

## Results

Out of 2923 patients in our database, altogether 1004 were eligible to calculate the frailty index by their parameters. Their median age was 69 (61–76,) years, their median FI was 0.36 (0.28–0.43). Overall, 929 (92.5%) patients were frail, 75 (7.5%) patients were in the non-frail group (≤ 0.210-), 271 (27%) patients in Frail-1 Group (0.211–0.310), and 658 (65.5%) in Frail-2 Group (> 0.311) (Table 2).

**Table 2:** Baseline clinical characteristics of patients by frailty groups

	All *n* = 1004	Non-frail *n* = 75	Frail 1 *n* = 271	Frail 2 *n* = 658	*p*-value
Age, years	69 (61–76)	63 (57–71)	66 (58–73)*	70 (64–76) + o	< 0.0001
Female, %	253 (25)	25 (33)	72 (27)	156 (24)	0.159
Physical status					
DBP Hgmm	73 (65–80)	75 (67–80)	75 (70–80)	72 (64–80)o	0.264
SBP Hgmm	125 (111–138)	121 (115–130)	120 (110–135)	130 (110–140) + o	0.076
PP Hgmm	50 (40–61)	47 (40–58)	48 (40–60)	53 (41–63) + o	0.004
BMI (819)	27.6 (24.7–30.9)	28.4 (23.9–30.3)	26.8 (24.5–30)	27.8 (24.8–31.2) + o	0.017
NYHA III/IV	559 (60)	25 (34)	140 (52)*	394 (60) + o	< 0.0001
Laboratory parameters					
BUN, mmol/L	8.1 (6.3–11.2)	5.7 (4.6–6.7)	6.9 (5.5–8.0)*	9.5 (7.3–12.9) + o	< 0.0001
Creatinine, μmol/L	100 (79–128)	77 (68–87)	87 (74–102)*	113 (89–147) + o	< 0.0001
Potassium, mmol/L	4.5 (4.2–4.9)	4.5 (4.1–4.8)	4.5 (4.2–4.8)	4.5 (4.2–4.9)	0.584
Sodium, mmol/L	138 (136–140)	138 (137–140)	139 (137–141)	138 (136–140) + o	0.006
Cholesterol, mmol/L	4.1 (3.3–5.1)	4.5 (3.8–5.2)	4.2 (3.6–5.2)	4 (3.2–5.1) +	0.006
HDL, mmol/L	1.17 (0.94–1.45)	1.19 (1.00–1.32)	1.16 (0.97–1.41)	1.18 (0.93–1.48)	0.953
Uric acid, μmol/L	404 (326–492)	364 (296–417)	382 (317–454)	432 (336–521) + o	< 0.0001
Hgb, g/dL	13.7 (12.4–14.8)	14 (13.2–14.7)	14 (12.9–15.1)	13.6 (12.2–14.7) + o	< 0.0001
PTL, G/L	203 (161–251)	229 (192–267)	213 (177–256)	194 (152–244) + o	< 0.0001
Ly, %	21 (16–26)	24 (19–29)	22 (18–27)	20 (15–26) + o	0.004
Medical history					
AF, %	395 (39)	13 (17)	67 (25)	315 (48) + o	< 0.0001
HT, %	785 (78)	40 (53)	188 (69)*	557 (85) + o	< 0.0001
MI, %	434 (43)	9 (12)	75 (28)*	305 (46) + o	< 0.0001
PCI/CABG, %	424 (42)	9 (12)	70 (26)*	345 (52) + o	< 0.0001
PAD, %	93 (9)	0 (0)	7 (3)	86 (13) + o	< 0.0001
Stroke, %	110 (11)	2 (3)	18 (7)	90 (14) + o	0.0004
DM, %	374 (37)	10 (13)	67 (25)*	297 (45) + o	< 0.0001
Cancer, %	85 (9)	5 (7)	18 (7)	62 (10)	0.307
COPD, %	114 (11)	2 (3)	251 (7)	122 (19) + o	< 0.0001
CIED, %	261 (26)	2 (3)	37 (14)*	222 (34) + o	< 0.0001
CKD, %	478 (48)	5 (7)	54 (20)*	419 (64) + o	< 0.0001
VT/VF, %	105 (11)	3 (4)	17 (6)	85 (13) + o	0.002
Echocardiographic parameters					
LVEDD, mm	63 (57–70)	58 (53–68)	64 (57–72)*	63 (58–70) +	0.029
LVESD, mm	53 (46–60)	47 (40–57)	55 (47–62)*	53 (47–60) +	0.003
LVEF, %	29 (24–33)	28 (21–38)	28 (24–33)*	29 (24–33) +	0.825
TAPSE, mm	19 (15–23)	22 (19–25)	20 (17–24)	18 (15–22) + o	< 0.0001
Frailty					
Frailty score	9.5 (7.5–12)	5 (4–5.5)	7.5 (7–8)*	11 (9.5–13) + o	< 0.0001
Frailty index	0.36 (0.28–0.43)	0.19 (0.15–0.20)	0.27 (0.25–0.29)*	0.40 (0.36–0.46) + o	< 0.0001

### Baseline clinical characteristics

In the different subgroups, the mean of age was associated with FI (r = 0.219396; *p* < 0.001) Compared to the non-frail group, frail patients were older [Median age was 63 (57–71) years in the Non frail and 70 (64–76) years in the Frail-2 group, *p* < 0.0001], more often men (67% vs. 76%; *p* = 0.159), and more likely to have cardiovascular and non-cardiovascular comorbidities: they had a higher uric acid (364 (296–417) vs. 432 (336–521) µmol/l, *p* < 0.001), creatinine (77 (68–87) vs. 113 (89–147) µmol/l, *p* < 0.001), and BUN (5.7 (4.6–6.7) vs. 9.5 (7.3–12.9) mmol/l, *p* < 0.001), levels, but lower hemoglobin (14 (13.2–14.7) vs. 13.6 (12.2.-14.7) g/dl, < 0.001), thrombocyte, (229 (192–267) vs. 194 (152–244) G/l, *p* < 0.001), and lymphocyte levels (24 (19–29) % vs 20 (15–26) %, *p* = 0.004). Patients with higher FI were more likely to have an ischemic etiology (12% vs. 52%, p < 0.001), worse NYHA functional class (NYHA III/IV: 34% vs. 60%, *p* < 0.001), and worse right ventricular function (TAPSE 22 (19–25) mm vs 18 (15–22) mm, *p* < 0.0001). They suffered more often from COPD (3% vs. 19%, *p* < 0.001), peripheral arterial disease (0% vs. 13%, *p* < 0.001), diabetes mellitus (13% vs. 45%, *p* < 0.001), and atrial fibrillation (17% vs. 48%, *p* < 0.001), than those with lower FI (Table 2).

### The use of optimal medical treatment and device therapy at baseline

In the proportion of those patients received optimal heart failure medical therapy was similar between the three groups. (OMT vs. Non-OMT *p* = 0.517). The proportion of CRT-D and CRT-P device proved to be equal in the different frail groups (Non-frail: 55 vs 44%, Frail 1: 58 vs 42%, Frail 2: 27% vs 20%, *p* = 0.907).

### Outcome data

#### Response to CRT by groups

When CRT response was investigated, FI was associated with a beneficial response, those in the non-frail group had a higher proportion of responders compared to Frail-2 group (79% vs. 58%, *p* = 0.044).

#### Primary endpoint

During the median follow-up time of 4.8 (2.3–7.2) years, a total of 640 (64%) patients reached the primary endpoint, 29 (39%) in the Non-frail group, 140 (52%) in Frail group 1, and 471 (72%) in the Frail group 2 (*p* < 0.001).

**Fig. 1:**
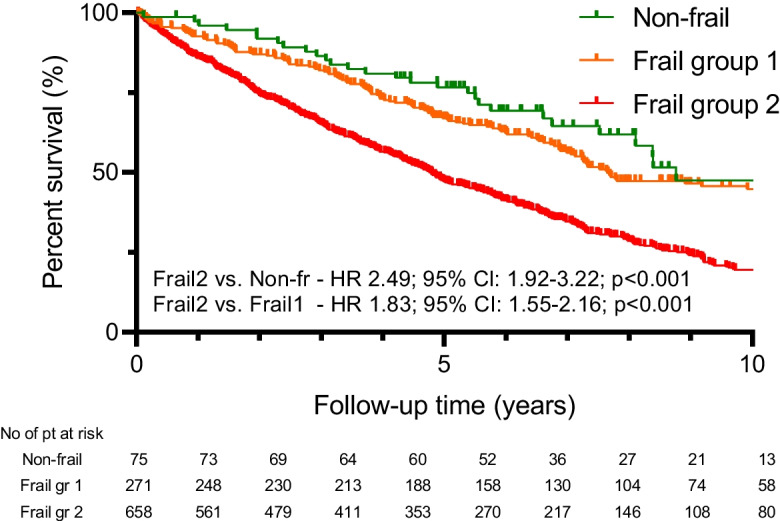
Kaplan–Meier estimate of all-cause mortality by failty groups

**Fig. 2:**
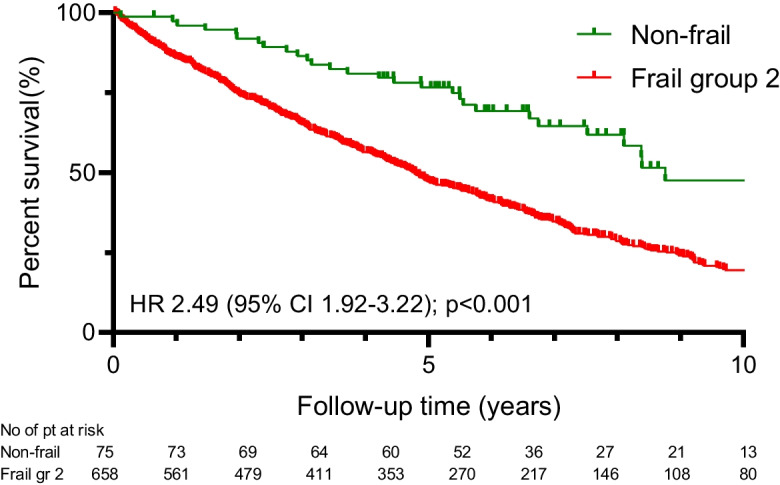
Kaplan–Meier estimate of all-cause mortality between patients in non-frail vs. Group Frail-2

**Fig. 3:**
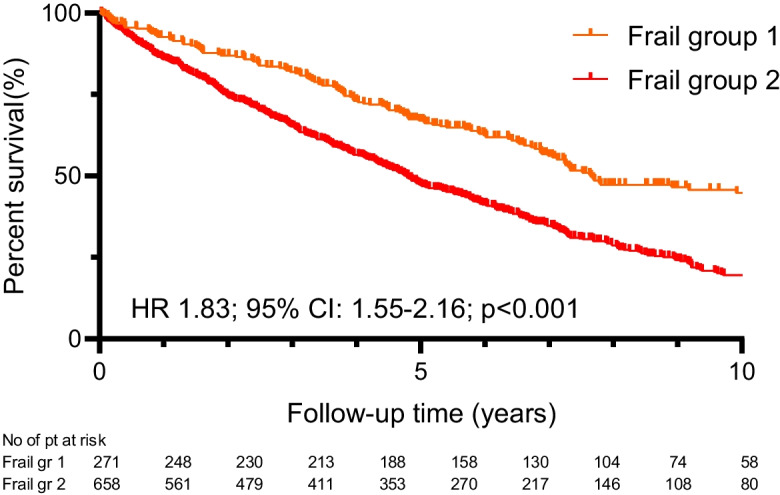
Kaplan–Meier estimate of all-cause mortality between patients in Group Frail-1 vs. Group Frail-2

The less favorable outcome could be observed in Group Frail-2 compared to the non-frail (HR 2.49, 95%CI 1.92–3.22; *p* < 0.001) (Figs. 1 and 2) and the Frail-1 group (1.83, 95%CI 1.55–2.16; *p* < 0.001) (Fig. 3).

## Discussion

In the current study of our large-scale, single-center, retrospective database, we demonstrated that in HFrEF patients who are eligible for CRT implantation, frailty is more frequent compared with previous HFrEF trials’ populations, affecting more than two third of our cohort. The calculated frailty index from clinically relevant covariates using the Rockwood cumulative deficit approach was correlated with the number of comorbidities and associated with an unfavorable outcome. Moreover, in those with greater frailty score, a less favorable treatment effect of CRT could be observed with a low rate of responders in both CRT-D and CRT-P patients. The proportion of CRT-D implantations was comparable in each group regardless of the frailty score, and in patients with higher frailty, the administration of optimal pharmacological treatment could be equally introduced.

Frailty and pre-frailty are very common in the heart failure population along the entire spectrum of the ejection fraction [18]. Due to the impaired heart function that leads to pathomechanisms affecting several organs, patients with heart failure have multiple times increased odds of being frail. However, age is linearly correlated with frailty, which is more frequent in the elderly, the impaired and systemic damage and the severity of heart failure also determine its prevalence.

Therefore, in the last decades, the terminology to characterize this condition has changed. As previously frailty phenotype was described as a biological syndrome with specific phenotypic presentations, nowadays frailty index incorporates the conditions of all deficits, most frequently used and calculated by Rockwood cumulative deficits approach.

Using this method, previous RCTs proved the ratio of frail patients and non-frail patients with lower than 0.21 FI lies between 38–50%, which is exceedingly high compared to our recent cohort. Moreover, patients with the highest FI higher than 0.311 ranged between 14–24%, which was 65.5% in our patient population, although the RCTs investigated a well-selected patient population, and precluded the enrollment of very high-risk patients [19].

In HFrEF cohorts those patients with higher FI are showing similar clinical characteristics; FI is correlated with older age and more severe HF symptoms [2]. Additionally, in our HFrEF cohort who were selected for a CRT device implantation, impaired renal function, anemia, atrial fibrillation, ischemic etiology, type II diabetes, and COPD were more frequent compared to less frail or non-frail patients. Moreover, when echocardiographic baseline parameters were assessed, those with the highest frailty had impaired right ventricular function as well showing a systemic, multiorgan impairment.

In line with these findings, the frailty score was described as a strong predictor of mortality. In the DELIVER trial, those with higher than 0.311 FI showed a 13.4-fold risk for mortality, which was a higher proportion than in our cohort, where patients with a FI higher than 0.311 showed a 2.5-fold risk for mortality [20]. In the analysis of PARADIGM and ATMOSPHERE studies, all-cause mortality and hospitalization from any cause occurred to have the strongest correlation with FI, showing that the assessment of FI incorporates the comorbidities and related risk of hospitalizations and mortality. Besides, patients with higher frailty complained of the worst quality of life, although after adding an effective treatment such as dapagliflozin, the improvement of QoL was the largest in the highest frailty group [8].

Sriwattanakomen et al. investigated an older CRT population with 469 HFrEF patients with age of > 75 years. During the mean follow-up period of 4.6 years, 82% of their patient died. In contrast with our results, they found that the choice of using a CRT-P was associated with higher FI and older age [21]. In our cohort, the rate of CRT-D was comparable in each FI group.

As a consequence of multiorgan failures and lower tolerance for pharmacological treatments, frail patients are less likely to receive new pharmacologic therapies and the occurrence of discontinuation of the optimal medical treatment is higher, and patients’ adherence could be lower [22]. However, in our recent study, those with the highest frailty received OMT in a comparable proportion regardless of the frailty. Despite such a balanced use of optimal pharmacological and device therapy, patients’ outcome already at a very early stage differed significantly, those with higher frailty failed to develop reverse remodeling, which was reflected in a less beneficial long-term outcome and high all-cause mortality rate.

Based on these results, early selection of frail patients in clinical practice is essential. Detecting those modifiable laboratory parameters (e.g. anemia, hyperuricemia, hyponatremia) and conditions (such as weight or BMI) that are described to correlate with unfavorable outcomes and, combined with a supervised exercise-based cardiac rehabilitation program might lead to a risk reduction in hospitalization or mortality in HFrEF patients [13]. Adding remote monitoring to the guideline directed medical therapy is associated with significantly improved clinical outcomes in patients with advanced HF [23] and could prevent frailty shift towards a worse status by the early detection of limited mobility [24]. Frailty is also proven to be a key factor in appropriate device selection. According to a recent study of Segar et al. baseline frailty modified the efficacy of ICD therapy with a significant mortality benefit observed among participants with HFrEF and a low frailty burden but not among those with a high frailty burden [25]. Additionally, those heart failure treatments, which showed a beneficial effect in decreasing cardiovascular mortality and heart failure events, should be used, their early administration is crucial in groups with higher frailty index.

### Limitations

Our study has a few limitations. First, this is a single-center, retrospective, observational study, may resulting in some imbalances in the study groups and data were missing for a small proportion of patients. As the enrollment period is 20 years, further imbalances in the treatment may influence our results. The single-center, retrospective design of the study may also limit the generalizability of findings to a broader population and could introduce selection bias.

## Conclusions

Based on our results, patients with heart failure and reduced ejection fraction, who are eligible for CRT implantation are exceedingly vulnerable with a high prevalence of frailty. The calculated frailty index was associated with higher rates of death from any cause and proved to be prevalent in individual risk stratification and can predict the outcome. Patients with high frailty index have a higher risk to fail to develop reverse remodeling despite having the same rate of optimal treatment both in terms of devices (such as CRT-D) and pharmacological therapy.
